# Otology in the South West
*Based on the Presidential Address to the Section of Otology of the Royal Society of Medicine, November, 1969.


**Published:** 1970-04

**Authors:** W. H. Bradbeer

**Affiliations:** Consultant Ear, Nose and Throat Surgeon, Torquay


### Otology in the South West *

**W. H. Bradbeer, M.B., M.S., D.L.O.**

Consultant Ear, Nose and Throat Surgeon, Torquay.

Otology in this country, together with laryngology, began to develop as a specialty in the middle of the 19th century under the influence of men like Yearsley and Toynbee, and later Sir Morel MacKenzie and Sir Felix Semon. They had a struggle to obtain recognition and the great German surgeon, Bilroth said of Otology — "It calls for a certain amount of heroism in a man to sacrifice himself to this therapeutically most thankless and limited phase in surgery".

The father of otolaryngology in the South-West was **Patrick Watson Williams** of Bristol. This great and historic city is famous for many things, including Cabot the explorer, the Zoo, Clifton Suspension Bridge, the Wills firm and family, Chatterton the poet, Bristol Milk, old glass, and last but not least its otolaryngologists. Watson-Williams was the son of a Bristol doctor and was born in Clifton in 1863. He was educated at Clifton College and the Bristol medical school and so was a real Bristolian. After training as a physician and obtaining his M.D., he was appointed as an assistant physician in 1890, and full physician in 1905. During this time he became interested in diseases of the Ear, Nose and Throat, and travelled up and down to London to work as a Clinical Assistant in the London Throat and Ear Hospital, and the Central London Throat Hospital. In 1906 he was put in charge of the newly formed Throat and Nose Department at the Bristol Royal Infirmary, to which was added the Ear Department in 1910. At first his surgical colleagues resented his claims to wield the knife, but later he achieved international reputation. He was especially interested in the diagnosis and treatment of chronic nasal sinusitis, about which he wrote extensively and devised a number of instruments, some of which are still used. In addition, he was an accomplished artist and illustrator. He took a very active part in fostering and developing his specialty and with his friend Sir Felix Semon, was a founder member of the Laryngological Society of London which subsequently became the Section of Laryngology of the Royal Society of Medicine. He was the President in 1910. He died in 1938 at the age of 75. One of his obituaries states—"He was a man of dignified and courtly presence, who won the confidence of his patients and colleagues by his dexterity and unremitting attention". His name is perpetuated in Bristol by the biennial Patrick Watson-Williams Memorial lecture.

The second otolaryngologist to be appointed in Bristol was the late **A. J. Wright.** He was another local product, having been born in Bristol and educated in the West Country at Blundell's School and the Bristol Medical School. He was the grandson of William Budd, F.R.S., who was one of the first to demonstrate that typhoid fever was due to an infectious organism. He was appointed surgeon-in-charge of the E.N.T. department at the Bristol General Hospital in 1913, a post which he filled with distinction until his retirement. I remember him as a spare, gaunt rather austere figure, but he was very kind, full of humour, and always ready to give practical and sensible advice to his juniors. He was especially interested in perceptive deafness and Ménière's disease, and devised a method of destroying the labyrinth by injecting alcohol with a needle pushed through the foot plate of the stapes. He was President of the Section of Otology in 1945-46, the Section of Laryngology in 1947-48, and Semon lecturer in 1949, a striking tribute to the high regard in which he was held by his contemporaries. I well remember his presidency of the Section of Otology because owing to the difficulties of accommodating the Summer meeting in post-war Bristol we were given the honour of staging it at Torquay and we look forward to a repeat performance next June. Jack Wright found time for an active public life and served on the Bristol City Council for nearly 20 years and was on numerous Hospital Committees. His hobbies were bird watching and sailing on the river Fal where he had a house at Feock. He was one of those people who never seem to grow old and it came as a shock when he died after a short illness in 1953 at the age of 70.

The third E.N.T. Surgeon to be appointed in Bristol was an Irishman called **J. P. I. Harty,** in 1921. Unfortunately, his health had suffered from war service and he died in 1928 at the early age of 47. He was a man of fine physique and had been a well-known Rugger player. Although he is largely forgotten, he has a special claim to our attention because J. Angell James said of him—"I was his dresser and later his house surgeon, and it was he who fired me with enthusiasm for E.N.T. work and gave me my basic training in it".

In the meantime another distinguished figure had entered the E.N.T. field in Bristol. This was **Eric Watson-Williams.** He had been made an assistant surgeon in his father's department in 1924. He was educated at Clifton, Caius College, King's College Hospital, and the Middlesex Hospital. He had served with distinction in

*Based on the Presidential Address to the Section of Otology of the Royal Society of Medicine, November, 1969.

the 1914-1918 war, being awarded the M.C. He was a man of wide education and culture, a connoisseur of good food and wine, and was a considerable classical scholar. He was an able surgeon and continued his father's work on the nasal sinuses. He made many contributions to medical literature and founded the S.W. Laryngological Association. On his retirement in 1955 he moved to Cambridge where he engaged himself in literary work and Greek translations until his death in 1964 at the age of 73.

Around about 1928 there were two further appointments in Bristol making a total of four surgeons. One of these was **Gordon Scarff** whom I got to know well as a fellow member of a Visiting Association. He was a very kindly but rather shy person and now lives in retirement in Sussex. The other appointment around this time was that of our illustrious friend, Jack Angell James who has been and still is a real guide, philosopher and friend to all of us in the South-West.

The present E.N.T. staff in Bristol includes five consultants. Besides the surgeons who have served otolaryngology so well in Bristol, there are of course many E.N.T. consultants scattered over the country who trained in Bristol and are a great credit to their school.

Next we go a little further west to visit **Weston-super-Mare.** Weston is a bright and breezy little town which has managed to retain some of the charm of an old fashioned sea-side watering place. There are donkeys on the beach and a pier with paddle steamers plying to and from South Wales. During the summer you will encounter numerous rather portly ladies and gentlemen wearing blazers and rubber-soled shoes, because this is a great bowling centre. The consultant here is keenly interested in the welfare and rehabilitation of the deaf, especially the elderly deaf. He has an excellent follow-up and after-care system for patients with hearing aids, largely run by voluntary help.

Proceeding further west, we come to **Taunton,** a traditional country market town. The E.N.T. Department here was founded by Mr. Graeme Allen just after the war, although there had been a G.P. specialist at the Hospital for some time before. Later on Mr. Allen was joined by another consultant and in 1954 a completely integrated E.N.T. unit was created at Musgrove Park—a former military hospital. Mr. Allen has recently retired.

It is only within the last ten years that North Devon has had its own otolaryngologist. He works at **Barnstaple,** another town with interesting historical associations, close links with Kipling's Stalkey & Co., and near the home of author Henry Williamson.

In Cornwall an E.N.T. Department was founded at **Truro** in 1936, and now has two consultants. Both of them have made interesting contributions to the literature. I would particularly like to draw attention to Mr. Michael Sheridan's advocacy of a closed radical mastoid operation, which he first described in 1943.

Coming back along the South Coast, we arrive at the port of **Plymouth** which is the largest city in Devon and another place with a great and romantic history. My enquiries into the beginning of otology here have brought to light what I think is a very remarkable fact. It is that the first E.N.T. Hospital was opened in Plymouth in 1889 and was in a large Victorian house near the present Greenbank Hospital. It had two six bedded wards and was run by a general practitioner called George Jackson, assisted by several other G.P.'s including Dr. Crowther who later became wholly engaged in E.N.T. work. The first fully trained otologist to be appointed in Plymouth was Mr. C. G. Prance of Bart's in 1929. By this time the E.N.T. hospital was much outdated and it was transferred to Greenbank in a new unit which is now bursting at the seams. Mr. Cyril Prance was joined after the war by Mr. R. Howarth. Both were interested in local politics and became chairmen of their Rural District Councils at Tavistock and Plympton. The present team in Plymouth consists of three consultants.

Next I would like to say something about life and otolaryngology in the **Torbay** area. The Department was started in 1913 by T. G. Fenton, who trained at St. Thomas' and was a great disciple of the late Walter Howarth. He looked exactly like Mr. Pickwick. After I had been in Torquay some time, I discovered that there had been some rather stormy events in the otological field which culminated in Fenton's resignation from the staff. He was succeeded by the late Cassidy de Wet Gibb. Gibb was a South African who learned his otology at Edinburgh and Guy's. After some years he left rather suddenly to take up an appointment in Gloucester, and Fenton was re-instated.

I arrived in Torquay in 1936 not long after the end of the Forsyte Saga and there were still a number of Forsyte-like characters in the town. The average wealthy Torquinian lived in a big Victorian Villa with several acres of ground and had a large domestic staff, a gardener and chauffeur, and often kept a yacht in the harbour maintained by a skipper. To begin with I lived and practised as a bachelor in a pleasant Georgian Terrace house and rather roughed it with only a housekeeper and manservant to look after me. In those days my income was less than £2,000 p.a. and I used to think that if I could earn £3,000 I would be able to own a yacht and perhaps even a Bentley. Later on I achieved a small yacht, but not the Bentley.

There was a great deal of entertaining in pre-war Torquay and one always dressed if asked out to dinner. The peak of the season was the Regatta fortnight, when there were still a number of J class yachts and other large craft competing. During the regatta there were numerous cocktail parties and at the foot of one's card it often said "Bleu et Blanc" which meant that one was expected to wear a yachting jacket and white trousers. In the autumn there was a County Ball at which the gentlemen wore tails and ladies elbow length gloves—and other things as well, of course.

This rather lotus life existence only lasted a few years and came to an end with the outbreak of war. During the war years I was kept pretty busy because many of the hotels in Torquay were taken over by the R.A.F. and we had some 10,000 R.A.F. recruits and, of course, many thousands of evacuee children to be looked after. A few months before 'D' day, the Americans descended on us. At that time my wife and I lived in a house with roomy basements and for some months we had 16 American G.I.'s billeted on us. They were a fine bunch of fellows and during this time our dogs and chickens 'never had it so good'.

One of my chief otological interests in recent years has been in the investigation and management of deaf children. I have become very much involved in this through the enthusiasm of one of our M.O.H.'s, Dr. Solomon. I would like to relate to you an amusing incident that happened recently at one of our follow-up clinics. A small boy of 7 had been asked to read a little story to us by the teacher of the deaf to demonstrate his progress. The story was about life on a farm. He was doing very well until he came to the phrase "and the farmer said, I'm bothered if I can catch that rabbit". When he reached the word "bothered" he used an alliterative word which is seldom heard in polite society. When admonished for this by his teacher the little boy who happened to be a farmer's son said—"Well that's what my Dad says when he can't catch one of his sheep".

We finally arrive at the lovely old city of **Exeter** which was my birthplace. The Royal Devon and Exeter Hospital was founded in 1741 and many of the original buildings are still in use, including the delightful board room which contains many interesting relics, including some splendid oil paintings of early worthies.

The first otologist in Exeter was **Robert Worthington,** surgeon, artist, and huntsman. Worthington was quite a remarkable character. He came from Yorkshire and was educated at Malvern, Rugby and Clare College, Cambridge. He trained as a doctor at the London Hospital, qualifying in 1904. At first he was mainly interested in pathology and became assistant director in the Department. However, he obtained his F.R.C.S. in 1909 and after spending some time abroad, where he took a special interest in all forms of endoscopy, he arrived in Exeter in 1911 as surgical registrar and assistant pathologist. In 1914 he was elected surgeon in charge of the E.N.T. department, where he worked for another thirty years. Many people found his manner rather intimidating, especially his patients. I think this was basically because he was rather shy and this in turn was due to the fact that all his life he was plagued by a stutter. There is an amusing anecdote about this. Someone sent Worthington a patient who stuttered, for advice. Worthington said to him—"I should l-l-like to s-s-send you to the m-m-m-man in L-London who c-c-c-cured me". Those who knew him well were aware that his apparent brusqueness hid an extremely kind and genial personality. Apart from his work, Worthington had two great interests—one was painting and the other hunting. He was a first class water colour artist with a special interest in Dartmoor. He usually took his paints with him when he followed hounds in case anything took his fancy after the meet. His last few years were rather tragic, but at the same time heroic. I would like to quote you a very moving passage written by my orthopaedic colleague, Mr. Norman Capener. "The war killed him without a doubt. He worked harder than ever, but it was the worst night of the blitz on Exeter during the "Baedeker" raids which tore him to pieces. In the basement of his house he had stored, because Exeter was thought relatively safe, many pictures by Bonnington and Gainsborough among other artists which were the property of his London friends. Early in the morning of May 4th, 1942, his house caught fire from an incendiary, and with superhuman detachment and no thought of personal safety he worked singlehanded like a demon emptying his basement of its treasures; moving them to places of safety and finding them exposed to new perils. He exhausted himself and yet continued to move and still more to move. His house eventually was destroyed, and with it he lost much of his own art collection and of his own work; but his friends and posterity were his first thought. Worthington never fully recovered from the shock of that night and after a series of premonitary episodes during the last eighteen months he died quietly from a severe haemorrhage on July 11th, 1945". Galsworthy lovers will see a striking resemblance between this vivid pen picture and Soames Forsyte's tragic end.

After Worthington's health failed his place on the staff was taken by Mr. W. M. Morris, who had been a consultant at the London Hospital. He had been serving in the R.A.F. and I knew him well during the war because he was stationed at the R.A.F. Officers' Hospital at the Palace Hotel, Torquay. Willie Morris was a man of very distinguished appearance and great personal charm. He was keenly interested in the surgery of the facial nerve.

When the war was over a second consultant post was established and in 1949 a third E.N.T. surgeon was appointed in Exeter. This was the late R. T. Hinde who was the most stimulating and congenial colleague and friend I have ever known. He had a delightful sense of humour and was a splendid mimic. However, he was a very serious minded young man and was extremely conscientious and dedicated to his work. Raymond learned his otology firstly at Guy's and later at Oxford under Ronald Macbeth and Gavin Livingstone. We were all quite desolated by his death in 1959 when he was only 43, following an operation for an acoustic neuroma.

This brings me to an end of our otological 'Cook's tour' of the S.W. during which we have looked a good deal into the past and glanced at the present. I would have liked to look into the future organisation of E.N.T. services, especially in my own area but I have some rather uonrthodox and revolutionary views which might get me into trouble, so I think it is as well for me to "keep mum".

I have gleaned the information for this talk from many sources, but I am especially grateful to Jack Angell James and his paper on the "Rise of Otolaryngology" which was his Presidential address to the Bristol Medico-Chirurgical Society in 1961.

**REFERENCE**

**James, J. Angell** "The Rise of Otorhinolaryngology" (1964). This Journal **79,** 26.

